# Supplementary material for: Inhibition of Rho-Associated Kinase Suppresses Medulloblastoma Growth
Source: Cancers (Basel). 2019 Dec 26;12(1):73. doi: 10.3390/cancers12010073 (PMC7016943; doi:10.3390/cancers12010073)
Supplement: Supplementary file 1 [file cancers-12-00073-s001.zip › cancers-6527667-final-supplementary/cancers-652767-proof -supplementary.docx]

Supplementary Materials

Inhibition of Rho-associated kinase suppresses medulloblastoma growth

Cecilia Dyberg, Teodora Andonova, Thale Kristin Olsen, Bertha Brodin, Marcel Kool, Per Kogner, John Inge Johnsen and Malin Wickström

**Table S1.** mRNA *ROCK2* expression in metastatic versus on metastatic samples, from the Cavalli data set (ref 20), using the R2 microarray analysis and visualization platform (<http://r2.amc.nl>). **Please view at the excel file.**

**Table S2.** Cancer cell lines used in the study. Abbreviations**:** Gr = group, MB = medulloblastoma, Shh = sonic hedgehog, sPNET = supratentorial primitive neuroectodermal tumor, Wt = wildtype.

| **Cell Line** | **Growth Pattern** | **Classification** | **Comment** |
| --- | --- | --- | --- |
| UW228-3 ^2^ | Adherent | MB Shh | *TP53* mutated |
| DAOY ^1^ | Adherent | MB Shh | *TP53* mutated |
| Med8a ^2^ | Suspension | MB Gr. 3 | *MYC* amplified |
| D425 ^2^ | Suspension | MB Gr. 3 | *MYC* amplified, *TP53* mutated |
| D458 ^2^ | Suspension | MB Gr. 3 | Recurrence from the same patient as D425; *MYC* amplified, *TP53* Wt |
| MB-LU-181 ^3^ | Neurospheres | MB Gr. 3 | *MYC* amplified |
| D283 ^1^ | Suspension | MB Gr. 3/4 |  |
| CHLA-01-MED ^1^ | Neurospheres | MB Gr. 4 | *MYC* amplified |
| CHLA-01R-MED ^1^ | Neurospheres | MB Gr. 4 | Recurrence from the same patient as CHLA-01-MED; *MYC* amplified |
| PFSK-1 ^1^ | Adherent | sPNET |  |
| MCF-5 ^1^ | Adherent | Fetal lung fibroblast |  |
| nHDF ^4^ | Adherent | Skin fibroblast |  |

^1^ Purchased from ATCC, USA; ^2^ A gift from Dr. M. Nistér, Karolinska Institutet (Stockholm) ^3^ Established, ref 42, ^4^ Purchased from PromoCell.

**Table S3.** IC_50_ (µM) for ROCK inhibitors RKI-1447, AT13148 and HA1077. IC_50_ values were determined from cell viability data from the WST-1 method. Cells were incubated for 72 h with increasing drug concentrations. All experiments were repeated at least three times. Abbreviations: Shh = sonic hedgehog, sPNET = supratentorial primitive neuroectodermal tumor.

| **Subgroup** | **Cell line** | **RKI-1447** | **AT13148** | **HA1077** |
| --- | --- | --- | --- | --- |
| Shh | UW228-3 | 23.6 µM | 14.1 µM | 115 µM |
|  | DAOY | 10.8 µM | 2.92 µM | 124 µM |
| Group 3 | D425 | 1.27 µM | 2.07 µM | 33.8 µM |
|  | D458 | 3.07 µM | 2.76 µM | 60.7 µM |
|  | Med8a | 1.08 µM | 1.03 µM | 44.8 µM |
|  | MB-LU-181 | 4.38 µM | 5.65 µM | 51.5 µM |
| Group 3/4 | D283 | 11.5 µM | 7.41 µM | 64.3 µM |
| Group 4 | CHLA-01-MED | 10.2 µM | 8.04 µM | 109 µM |
|  | CHLA-01R-MED | 4.59 µM | 6.62 µM | 67.7 µM |
| sPNET | PFSK-1 | 2.16 µM | 2.86 µM | 72.2 µM |
| Fibroblasts | MRC-5 | 15.5 µM | 7.86 µM | 78.7 µM |
|  | nHDF | 14.0 µM | 8.22 µM | 122 µM |

**Table S4.** (**A**): Significantly differently downregulated genes after RKI-1447 1 µM in D425, compared to vehicle treatment. (**B**) Significantly differently upregulated genes after RKI-1447 1 µM in D425, compared to vehicle treatment. **Please view at the excel file.**

**Table S5.** Top twelve most significantly downregulated Reactome terms/pathways. NES = C. Enrichments were considered significant if false discovery rate (FDR) < 0.25. Nom = nominal.

|  | **Downregulated REAC Gene Set** | **NES** | **FDR *q* Value** | **Nom *p***  **Value** | **Gene Set Identifier** |
| --- | --- | --- | --- | --- | --- |
| 1 | GPVI mediated activation cascade | −1.76 | 0.163 | <0.001 | R-HSA-114604 |
| 2 | Peptide chain elongation | −1.75 | 0.110 | <0.001 | R-HSA-156902 |
| 3 | Influenza viral RNA transcription and replication | −1.74 | 0.079 | <0.001 | R-HSA-168273 |
| 4 | SEMA4D induced cell migration and growth cone collapse | −1.73 | 0.085 | <0.001 | R-HSA-416572 |
| 5 | SEMA4D in semaphoring signaling | −1.72 | 0.080 | <0.001 | R-HSA-400685 |
| 6 | Semaphorin interactions | −1.70 | 0.097 | 0.001 | R-HSA-373755 |
| 7 | Interferon gamma signaling | −1.70 | 0.090 | 0.005 | R-HSA-877300 |
| 8 | Signaling by Rho GTPases | −1.66 | 0.146 | 0.001 | R-HSA-194315 |
| 9 | 3-UTR mediated translational regulation | −1.65 | 0.147 | 0.005 | REACT_1762 |
| 10 | Nonsense mediated decay enhanced by the exon junction complex | −1.65 | 0.135 | 0.004 | R-HSA-975957 |
| 11 | G alpha (12/13) signaling events | −1.65 | 0.135 | 0.006 | R-HSA-416482 |
| 12 | SRP dependent cotranslational protein targeting | −1.64 | 0.126 | <0.001 | R-HSA-1799339 |

**Table S6.** Antibodies used in the study. Abbreviations: CST = Cell Signaling Technology, IHC = immunohistochemistry, SCBT = Santa Cruz Biotechnologies, WB = western blot.

| **Antibody** | **Primary Ab Dilution** | **Host Species** | **Company** | **Catalog Number** | **Size** | **Application** |
| --- | --- | --- | --- | --- | --- | --- |
| MLC2 | 1:500 | Rabbit | CST^1^ | 3672 | 18 kDa | WB |
| pMLC2(Ser19) | 1:1000 | Rabbit | CST | 3671 | 18 kDa | WB |
| RHOA | 1:200 | Mouse | SCBT^2^ | SC418 | 20 kDa | WB |
| RHOB | 1:1000 | Rabbit | CST | 63876 (D1J9V) | 21 kDa | WB |
| JUN | 1:1000 | Rabbit | CST | 9165 (60A8) | 40-50 kDa | WB |
| ROCK1 | 1:1000 | Rabbit | CST | 4035 | 160 kDa | WB |
| ROCK2 | 1:1000 | Rabbit | CST | 9020 | 160 kDa | WB |
| Vimentin | 1:1000 | Rabbit | CST | 5741 (D21H3) | 57 kDa | WB |
| GAPDH | 1:10000 | Mouse | Abcam^3^ | 8245 | 37 kDa | WB |
| Vinculin | 1:10000 | Rabbit | Abcam | EPR8185 | 124 kDa | WB |
| Ki67 | 1:800 | Rabbit | DAKO^4^ | A0047 | − | IHC |

^1^ Cell Signaling Technology, Leiden, The Netherlands, ^2^ Santa Cruz Biotechnologies, Heidelberg, Germany, ^3^ Abcam, Cambridge, UK, ^4^Dako, Agilent Technologies, Inc., Santa Clara, CA, USA.

**Figure S1.** mRNA expression on *ROCK1* and *ROCK2* in cell lines used in our study and available in the Cell Line Encyclopedia (Broad Institute) and densitometric analyses of protein expression corresponding to Figure 1D. (**A**). mRNA expression (2 log) of *ROCK1* and *ROCK2* from medulloblastoma cell lines used in our study and available in Cell Line Encyclopedia (Broad Institute, DepMap Public 19Q3: https://depmap.org/portal/download/all/?release=DepMap+Public+19Q3&file=CCLE_expression.csv). (**B**–**C**). Protein expression was assessed by western blot with antibodies for ROCK1 (160 kDa), ROCK2 (160 kDa) and loading control vinculin (124 kDa) (**B**) and for phosphorylated MLC2 (Ser19) (18 kDa) compared to total MLC2 (18 kDa) (**C**) in nine different medulloblastoma cell lines.

**Figure S2.** Densitometric analyses of protein expression corresponding to Figure 3A and C. (**A**, **B**) RKI-1447 inhibited downstream signaling in the Rho/ROCK pathway assessed by protein expression of phosphorylated MLC (Ser19) (18 kDa) compared to total MLC (18 kDa) and loading control GAPDH (37 kDa) after RKI-1447 incubation for 2 h (**A**) and 48 h (**B**). Protein expression was investigated in Med8a (1 µM RKI-1447), DAOY (10 µM RKI-1447), D425 (1 µM RKI-1447) and D458 (3 µM RKI-1447) with western blot. (**C**) The siRNA knockdowns of *ROCK2* were confirmed with western blotting on protein expression of ROCK2 (160 kDa) with GAPDH (37 kDa) as loading control at 72 h in D425 and DAOY cells.

**Figure S3.** Cell morphology of UW228-3 after RKI-1447 incubation. Morphological elongation was evident in UW228-3 in response to RKI-1447 (20 μM, 24 h and 72 h). Images were acquired using a phase contrast microscope (acquired at 20X).

**Figure S4.** Size of wound at start in the assessment of RKI-1447 on medulloblastoma migration, measured by wound assay. There were no differences between the different treatments in wound size at time 0 h (one-way ANOVA, DAOY: *p* = 0.81 and UW-228-3: *p* = 0.81). Four images were acquired per treatment and the experiment was repeated three time for DAOY and four times for UW228-3. Values displayed are mean with S.D.

**Figure S5.** RKI-1447 (10 µM) repressed the invasion ability in medulloblastoma cells. Real-time invasion analysis after treatment with 10 µM RKI-1447 in DAOY cells in transwell cell chambers coated with 15% matrigel, monitored in the xCELLigence RTCA system (two-way ANOVA with Bonferroni post-test, *p* = 0.021 for treatment over the 40 h period, *p* < 0.05 from 23 h and forward for control vs 10 µM, *p* = 0.0002 at 40 h). Mean with S.E.M. of three independent experiments are shown.

**Figure S6.** Densitometric analyses of protein expression corresponding to Figure 5C, D. RKI-1447 downregulate Rho, Jun and vimentin. Western blot analysis on ROCK1 (160 kDa), ROCK2 (160 kDa), RHOA (21 kDa), RHOB (21 kDa), JUN (40-50 kDa), vimentin (53 kDa), and loading control GAPDH (26 kDa) or vinculin (124 kDa) protein expression in D425 (**A**, **B**, **C**) or Med8a (**D**) cells treated with 1 µM RKI-1447.

**Figure S7.** Weight gain of NMRI nu/nu mice treated with RKI-1447 dihydrochloride (2H_2_0) (80 mg/kg) by a daily intraperitoneal injection (*n* = 11) compared to controls (*n* = 10). A transient weight loss was evident, at day 4–5 the RKI-1447 treated mice showed a significantly less weight gain compared to control mice (multiple t-tests day 1 to 10 gave the following adjusted P values: day 1 *p* =0.41, day 2 *p* = 0.41, day 3 *p* = 0.060, day 4 *p* = 0.013, day 5 *p* = 0.043, day 6 *p* = 0.19, day 7 *p* = 0.41, day 8 *p* = 0.40, day 9 *p* = 0.19 and day 10 *p* = 0.10). Mean and S.D. of weight gain compared to body weight at inclusion are presented.

**Full blots for all western blot analyses.**

| 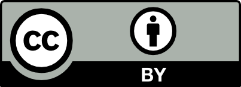 | © 2019 by the authors. Licensee MDPI, Basel, Switzerland. This article is an open access article distributed under the terms and conditions of the Creative Commons Attribution (CC BY) license (http://creativecommons.org/licenses/by/4.0/). |
| --- | --- |
